# Human Motor Thalamus Reconstructed in 3D from Continuous Sagittal Sections with Identified Subcortical Afferent Territories

**DOI:** 10.1523/ENEURO.0060-18.2018

**Published:** 2018-07-04

**Authors:** Igor Ilinsky, Andreas Horn, Perrine Paul-Gilloteaux, Pierre Gressens, Catherine Verney, Kristy Kultas-Ilinsky

**Affiliations:** 1 Unité 1141 Institut National de la Santé et de la Recherche Médicale, UMRS 1141, Hôpital Robert Debré, Paris, Université Paris Diderot, Sorbonne Paris Cité, Paris France; 2 Movement Disorders and Neuromodulation Unit, Charité–University Medicine, Berlin 10967, Germany; 3 Unité Mixte de Recherche 144, PICT IBISA Institute Curie, Centre National de la Recherche Scientifique, Paris 75005, France; 4 The University of Iowa, Iowa City, IA 52242

**Keywords:** brain imaging, cerebellar and basal ganglia-thalamic connections, human thalamic nomenclature, MNI space, stereotactic maps, VIM

## Abstract

Classification and delineation of the motor-related nuclei in the human thalamus have been the focus of numerous discussions for a long time. Difficulties in finding consensus have for the most part been caused by paucity of direct experimental data on connections of individual nuclear entities. Kultas-Ilinsky et al. (2011) showed that distribution of glutamic acid decarboxylase isoform 65 (GAD65), the enzyme that synthesizes inhibitory neurotransmitter γ-aminobutyric acid, is a reliable marker that allows to delineate connectionally distinct nuclei in the human motor thalamus, namely the territories innervated by nigral, pallidal, and cerebellar afferents. We compared those immunocytochemical staining patterns with underlying cytoarchitecture and used the latter to outline the three afferent territories in a continuous series of sagittal Nissl-stained sections of the human thalamus. The 3D volume reconstructed from the outlines was placed in the Talairach stereotactic coordinate system relative to the intercommissural line and sectioned in three stereotactic planes to produce color-coded nuclear maps. This 3D coordinate-based atlas was coregistered to the Montreal Neurological Institute (MNI-152) space. The current report proposes a simplified nomenclature of the motor-related thalamic nuclei, presents images of selected histological sections and stereotactic maps illustrating topographic relationships of these nuclei as well as their relationship with adjacent somatosensory afferent region. The data are useful in different applications such as functional MRI and diffusion tractography. The 3D dataset is publicly available under an open license and can also be applicable in clinical interventions in the thalamus.

## Significance Statement

This is the first demonstration of revised maps of human motor thalamic nuclei that clearly identify subterritories based on their afferents in three stereotactic planes derived from a continuous series of histologic sections from a single brain. Consistent interslice distance and high resolution ensure a high reliability of target coordinates because the dataset is applicable in all planes. Nuclear outlines based on a specific marker and straightforward modified nomenclature reflect the functional and connectional properties of motor-related regions. 3D coordinate-based atlas space is coregistered with the most modern version of the widely used MNI space (multispectral MNI-152 2009b NLIN Asym template). The report introduces a unique tool for researchers and clinicians studying the human thalamus with experimental and imaging techniques.

## Introduction

Longstanding controversy regarding nomenclature and delineations of so-called motor thalamic nuclei, i.e., the regions receiving basal ganglia and cerebellar input, in the human brain remains unresolved despite numerous attempts undertaken during the last decades and substantial progress achieved toward it recently (Kultas-Ilinsky et al., 2011; for review, see Mai and Forutan, 2012).

Notwithstanding its conjectural importance, the issue became a focus of attention of stereotactic neurosurgeons in the middle of the last century when certain regions of the human motor thalamus became targets of neurosurgical interventions in treatment of movement disorders.

Traditionally, clinical neurologists use nomenclature by Hassler (Schaltenbrandt and Bailey, 1959; Schaltenbrandt and Wahren, 1977), by which the human motor thalamus consists of a great number of small cytoarchitectonic entities with unidentified functional significance and connectional specificity, which, at that time, were not as well understood as at present. At the same time, clinical observations demonstrated that lesions in two Hassler’s subdivisions, Vim and Vop, eliminated or significantly reduced parkinsonian tremor. It was suggested that cerebellothalamic fibers pass through and/or terminate in these regions but an involvement of pallidothalamic projections was also suspected (Hassler et al., 1979).

In contrast, experimental neuroscientists working with nonhuman primates preferred to use the thalamic nomenclature by Walker (1938) that was adopted by Olszewski (1952) in stereotactic atlas of *Macaca mulatta* by which the motor thalamus consisted of several, but substantially fewer than Hassler’s, subdivisions of three nuclei, ventral anterior (VA), ventral lateral (VL), and a part of ventral posterior (VP) connected with premotor and primary motor cortices.

Since then, a large body of data has accumulated on cortical and subcortical connections of the thalamus in pathway tracing studies in nonhuman primates and other species. These data as well as studies of various immunohistochemical staining patterns led to numerous revisions of human and nonhuman thalamic nomenclatures and parcellations (Ilinsky et al., 1985**;** Ilinsky and Kultas-Ilinsky, 1987, 2002a, 2002b; Hirai and Jones, 1989; Stepniewska et al., 1994; Percheron et al., 1996; Macchi and Jones, 1997; Morel et al., 1997; Kultas-Ilinsky et al., 2003, 2011; Krack et al., 2002; Calzavara et al., 2005; Jones, 2007, Mai et al., 2008). Nonetheless, an absence of a continuous series of cytoarchitectonic plates illustrating topographic relationships of revised subdivisions (with the exception of the atlases of the thalamus of *M. mulatta* by Ilinsky and Kultas-Ilinsky, 2002b; and *Macaca fascicularis* by Lanciego and Vázquez, 2012) has impeded a wide acceptance of proposed modifications. It is especially true with respect to the human thalamus where delineations of the nuclei, and particularly those of the most controversial motor-related subdivisions, have for the most part remained unchanged. Technical limitations involved in the work with human brain tissue and the absence of direct experimental data on distribution of subcortical afferents are the main reasons.

Recently, it was demonstrated that distribution patterns of glutamic acid decarboxylase isoform 65 (GAD65) in monkey and human thalami were remarkably similar, if not identical (Kultas-Ilinsky et al., 2011). Earlier numerous light and electron microscopic studies in nonhuman primates (for review, see Ilinsky and Kultas-Ilinsky, 2001; Kultas-Ilinsky and Ilinsky, 2001) have demonstrated that thalamic territories of distribution of nigral, pallidal, and cerebellar afferents differ by types and combinations of GABA-ergic fiber and cellular components present, and this results in GAD65 staining patterns that are specific for each territory. Thus, the immunocytochemical staining for GAD65 in the human thalamus provided an indirect but reliable means for identification of extent of projection zones of nigral, pallidal, and cerebellar afferents in this species.

In the present study, we used the distribution patterns of GAD65 from Kultas-Ilinsky et al. (2011) with underlying cytoarchitectonic features of the areas to outline the three subcortical afferent territories of the human motor thalamus in continuous series of Nissl-stained sagittal sections. These outlines were color-coded and used for 3D reconstruction of the thalamus. The resulting volume was sectioned in three stereotactic planes based on the line between anterior and posterior commissures to provide continuous maps of all thalamic nuclei. Finally, the same 3D volume was coregistered to the MNI space, which serves as basis for neuroimaging studies, to broaden the application options. This report demonstrates selected images of computer processed histologic sagittal sections containing motor thalamic nuclei, corresponding color-coded maps showing these nuclei in three stereotactic coordinate planes, and their placement within MNI space. The entire atlas and volumetric MNI version of the dataset are available at www.humanmotorthalamus.com. Moreover, the MNI version integrated into an open source deep brain stimulation analysis software is available at www.lead-dbs.org (Horn and Kühn, 2015).

## Materials and Methods

Four postmortem human brain specimens (two males and two females) perfused with 0.1 M phosphate buffer followed by 4% paraformaldehyde in the same buffer used in this study were obtained from The University of Iowa Department of Anatomy Deeded Body Program.

Treatment of the human tissue was in complete compliance with the guidelines of the Institutional Review Board and Human Subjects Office of the University of Iowa.

### Histologic tissue processing

Thalami with adjacent parts of midbrain and basal ganglia from both hemispheres were dissected out and postfixed in fresh fixative, 4% paraformaldehyde in 0.1 M phosphate buffer (the same fixative that was used for perfusion), for a period from one to two years in a cold room. Before sectioning thalamic blocks were placed on a leveled horizontal surface. A hypodermic needle with a rounded sharp tip attached to a syringe, which was fixed in a stereotactic holder positioned strictly perpendicular to the horizontal surface, was driven through the anterior (AC) and posterior (PC) commissures as they appear in the midsagittal cut to establish fiducial marks in the tissue blocks. Sagittal sections were cut on a freezing microtome MM France (Microm Microtech France) at 50-µm thickness in the plane parallel to the midsagittal plane. All sections were collected and placed in individual numbered compartments to maintain the sequence. Entire section series was mounted on slides, stained with thionin and used for analysis of cytoarchitecture. For imaging and 3D reconstruction, one series of sections through the thalamus of one female brain was chosen. Criteria for choice were the quality of sections and tissue preservation, completeness of the section series, as well as the length of the AC-PC line, i.e., intercommissural line, of 23 mm. This length was comparable to that of the brain used for demonstration of sagittal sections in the Schaltenbrand and Bailey atlas (23.5 mm) thus facilitating comparisons of coordinates of different structures in the two datasets.

The choice of the sagittal section plane was determined by the following considerations. (1) It is easier to maintain the consistency of cutting angle when sectioning in sagittal plane as compared to coronal and horizontal; this, in turn, allows more accurate comparisons of different brains. (2) According to neuroanatomical data, the projection zones of major subcortical afferent systems to the thalamus are arranged in anterior-posterior sequence, hence their topographical relationships are better appreciated in sagittal plane. (3) Landmarks of the coordinate system, i.e., positions of AC and PC in the midsagittal plane, can be easily marked and their projections followed in sagittal sections. (4) Total number of sagittal sections is significantly smaller than that of more frequently used coronal sections.

### Image analysis and 3D reconstruction

**Figure 1.**
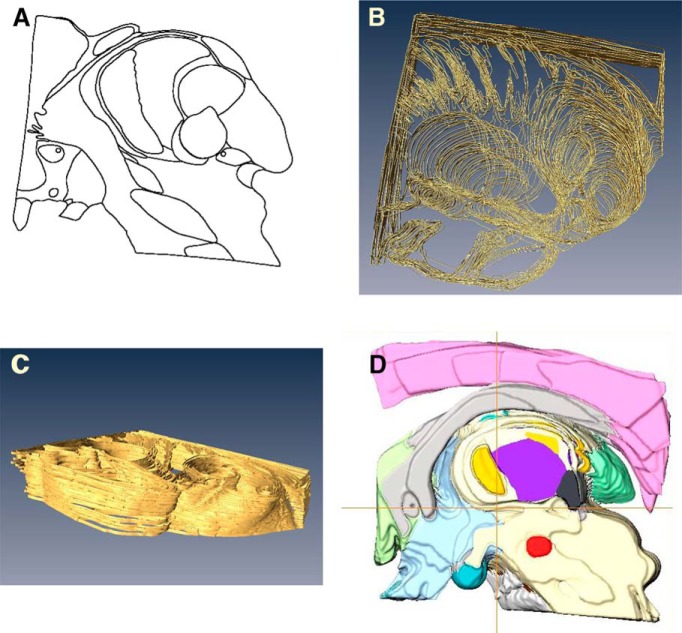
Illustration of consecutive stages of image processing. ***A***, Example of nuclear outlines traced from a 50-µm-thick section. ***B***, Example of a group of aligned outlines. ***C***, A group of outlines with thickness added. ***D***, Medial view of 3D-reconstructed volume of color-coded structures. Positions of coordinate planes are indicated with the two perpendiculars: horizontal axis indicates position of the intercommissural plane, i.e., zero horizontal plane, vertical axis shows the position of the zero frontal plane.

**Figure 2.**
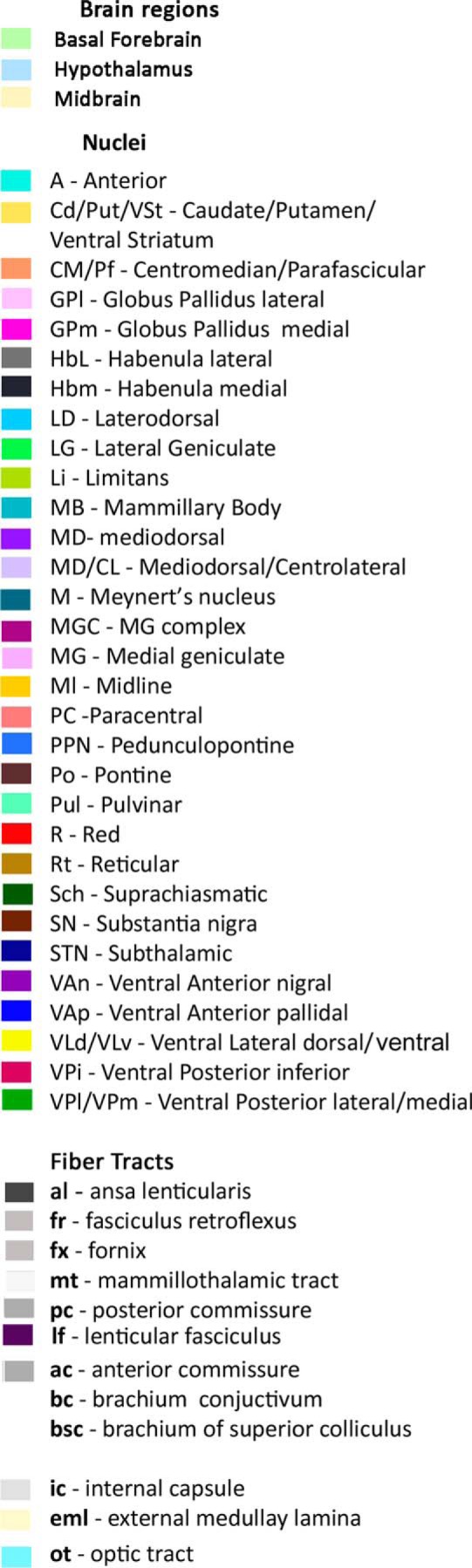
Color code and nuclear abbreviations.

All 50-μm-thick Nissl-stained sections from the chosen tissue block were photographed with a Sony digital camera. Resulting digital images were processed in Photoshop (Adobe Systems Incorporated) correcting the brightness and contrast and removing specks of dust and debris. Nuclear outlines were identified in an enlarged image of one section from each group of five sections and verified under microscope comparing with GAD65 staining pattern. Then the outlines of the sections and nuclei in them were retraced (Fig. 1*A*) in Adobe Illustrator (Adobe Systems Incorporated) and aligned (Fig. 1*B*) using fiducial marks. The set of aligned outlines was then imported as an image sequence (Rasband, 1997–2014) to ImageJ (https://imagej.nih.gov/ij/), stacked, and binarized by simple thresholding. The outlines were closed using mathematical morphology operation called dilation to make the outlines thicker and closed and transferred to ImageJ where they were filled with varying colors (Fig. 2).

These color-coded images were used for 3D reconstruction. Processing was done with Amira software (FEI Software). Stacks were loaded as a 3D volume in the physical space calibrated in millimeters based on the 250-µm section thickness and the pixel size derived from the original length of intercommissural line measured from the posterior end of AC to anterior end of PC in one of medial sections, in which the needle tracks were perfectly parallel and the outlines of the commissure markings clear. Each structure was segmented by successive 3D thresholding of each color, slightly smoothed, and converted to a 3D surface (Fig. 1*C*,*D*). The volume composed of all surfaces was rotated manually under Amira to align it within Cartesian coordinate planes in Amira physical space. The latter was made visible by the creation of a reference stacks containing axes created under MATLAB (MathWorks). Finally, millimeter graduations were placed on the axes.

Volumes in cubic millimeters (Table 1) were extracted using the material measurements of Amira directly from the 3D segmented labels and computed as the number of voxels in each volume of interest multiplied by the volume of one voxel in cubic millimeters.

**Table 1.** Volumes of human motor-related thalamic nuclei and sources of their afferent inputs

Nuclei	Volume in mm^3^
	
VAn	68.38
VAp	457.15
VL (VLd andVLv)	843.59
Substantia nigra	344.65
Medial globus pallidus	495. 81
Deep cerebellar nuclei	Not available

**Figure 3.**
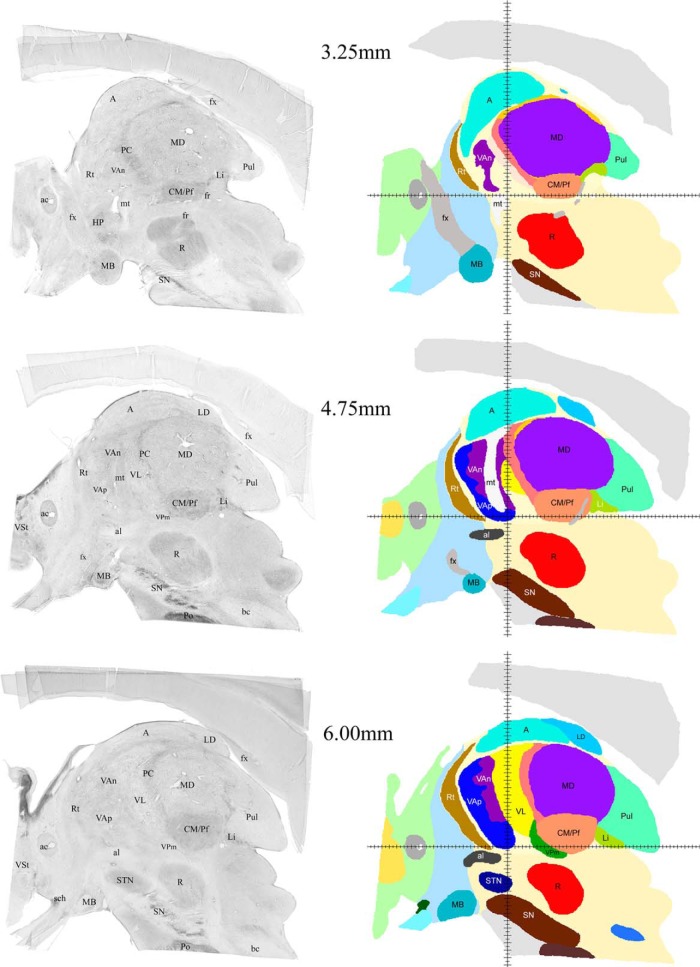
Examples of sagittal histologic images and color-coded maps from 3.25 to 6 mm from the midline. To illustrate the medio-lateral extent of the three motor nuclei this and the following four figures (Figs. 4–7) show selected images of the series of sagittal computerprocessed Nissl-stained sections (left column) and corresponding sagittal cuts of reconstructed 3D volume with color-coded nuclei (right column) at different distances from the midline. The distances of each pair of images from the midline are indicated in the middle. VAn, dark purple; VAp, dark blue; VL, bright yellow; al, ansa lenticularis; fx, fornix; mt, mammillothalamic tract. For labeling and abbreviations of all other structures, see Figure 2.

**Figure 4.**
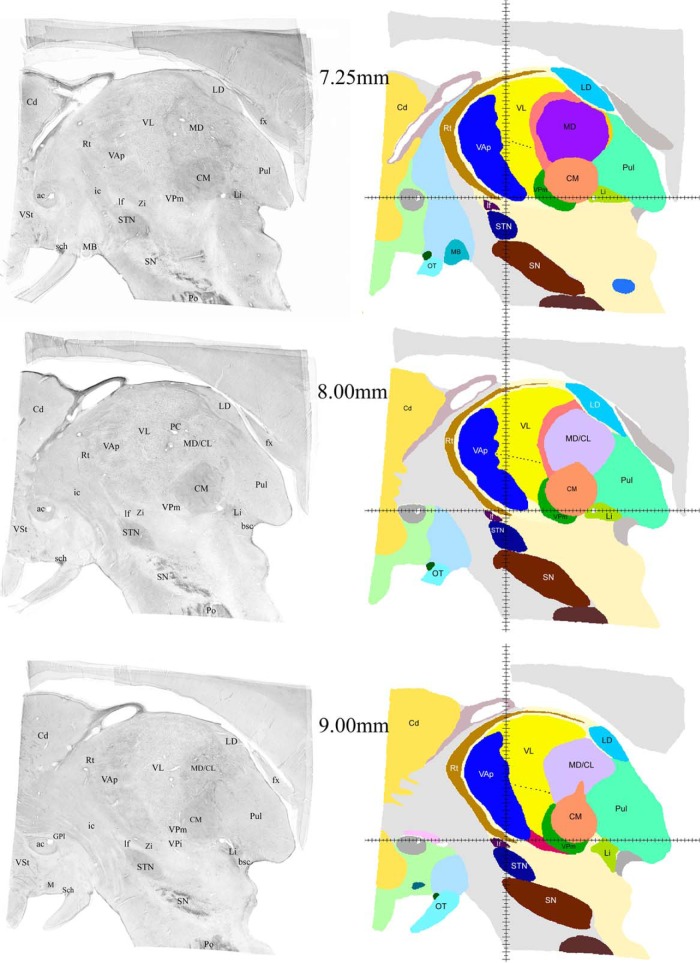
Examples of sagittal histologic images and color-coded maps from 7.25 to 9 mm from the midline. Continuation of the series shown in Figure 3. The black dashed contour in VL indicates the approximate dorsal boundary of VLv. Groups of cells in adjacent motor nuclei reach significantly into each other territories making the boundary between them very uneven. Despite the low contrast this can be noticed in histologic images in the left column. These zigzags are smoothed in 3D volume due to technical limitations. Zi, zona incerta; lf, lenticular fasciculus, the rest of the labeling as in Figure 3.

**Figure 5.**
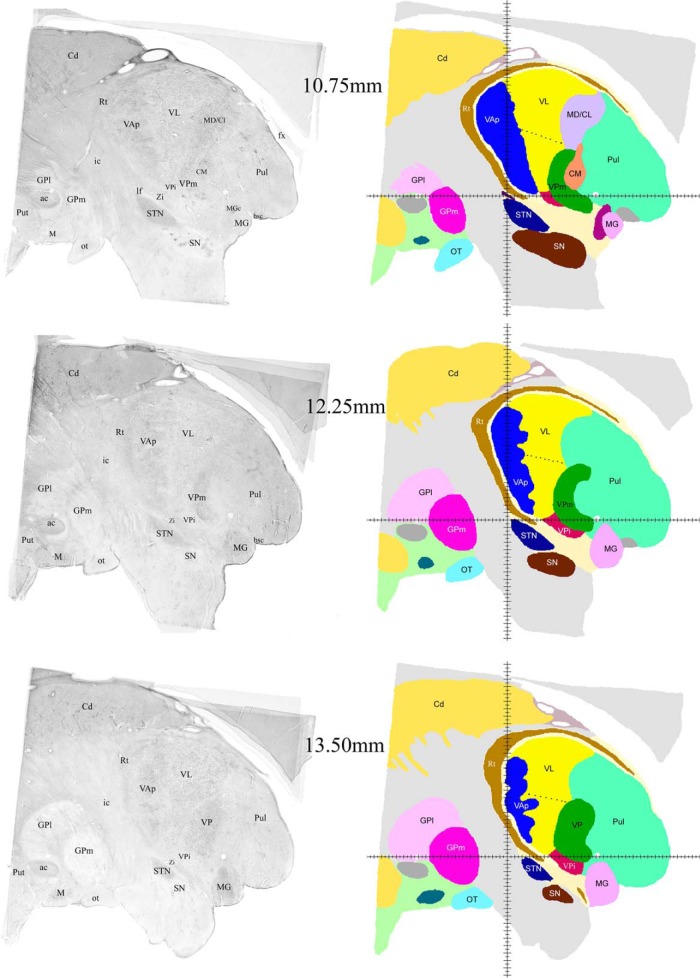
Examples of sagittal histologic images and color-coded maps from 10.75 to 13.5 mm from the midline. Continuation of the series shown in Figures 3 and 4. Labeling as in Figures 3 and 4.

**Figure 6.**
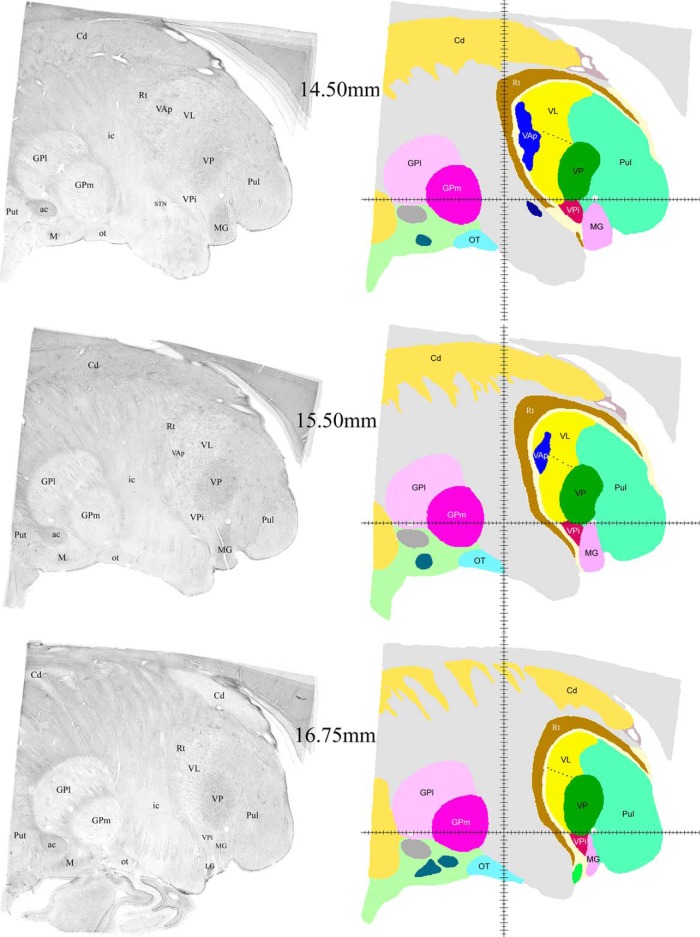
Examples of sagittal histologic images and color-coded maps from 14.5 to 16.75 mm from the midline. Continuation of the series shown in Figures 3–5. Labeling as in Figures 3–5.

**Figure 7.**
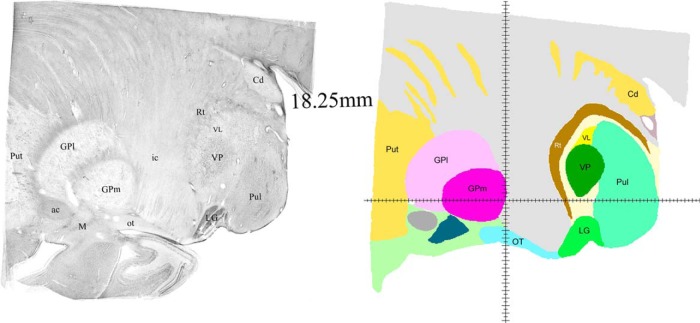
Sagittal histologic images and color-coded map at 18.5 mm from the midline. The most lateral extension of VL. Labeling as in Figures 3–6.

To illustrate histology, images of each group of five adjacent sections were merged in Adobe Photoshop resulting in 250-µm-thick digital sections in which general outlines of major nuclei became more evident. The latter were further emphasized using contrast and brightness adjustments. Since the quality of the available human brain tissue was not perfect, some defects during cutting were unavoidable. Merging adjacent sections helped overcome these defects and rebuild a representative image for each 250 μm (Figs. 3–7, left column).

The coordinate system used was based on the intercommissural plane, i.e., zero horizontal plane that passes through intercommissural line perpendicular to the midsagittal plane. A plane perpendicular to the zero horizontal plane passing through the midpoint of AC-PC line established the zero coronal plane. Distances of coronal sections posterior to the zero coronal plane and horizontal sections below the zero horizontal plane were marked with negative numbers. Mediolateral distances were measured from the midsagittal plane, i.e., midline, and marked accordingly.

### Reconstruction of 3D version and registration to MNI space

To reconstruct a 3D version of the human motor thalamus atlas that may be nonlinearly deformed to subject-/patient-specific anatomy in both neuroimaging and surgical applications, labels derived from stacks of histology-based sagittal maps were digitized and converted to a volume in NIfTI-1 format. A coronal T2-weighted MRI scan of the histologic stacks was acquired and equally converted to NIfTI-1 format. Histology-informed labels and T2-weighted MRI blocks were linearly coregistered (6-DOF) using 3D Slicer software (www.slicer.org). Both were then manually coregistered to the MNI-152 2009b NLIN ASYM normative brain template (Fonov et al., 2009) using the same 12-DOF affine transform. To refine precision, in the first step the MRI block was nonlinearly registered to the T2-weighted version of the MNI template using Advanced Normalization Tools (ANTs; http://stnava.github.io/ANTs/; Avants et al., 2008) as implemented in the “effective (low variance)” preset in Lead-DBS software (www.lead-dbs.org; Horn and Kühn, 2015). The nonlinear deformation was then applied to the histology-informed labels using ITK Generic Label interpolation as implemented in ANTs. In the second refinement step, the pseudo-MRI registration approach introduced and validated by Chakravarty et al. (2006, 2008) was applied. Specifics of the multispectral multi-iterative version of the approach applied here are described in detail elsewhere (Ewert et al., 2018). Briefly, intensity-matched pseudo-MRIs were created from histology-derived labels by assigning each label the average intensity under its volume defined in the T1-, T2-, and PD-weighted MNI templates. This resulted in pseudo-MRIs that appeared similar to the T1-, T2-, and PD-weighted MRI-templates, respectively. Again, using the same ANTs preset mentioned above, these labels were further refined to conform to template space in a multispectral fashion, i.e., pairing the pseudo-T1 with the T1-MNI template, the pseudo-T2 with MNI-T2 and pseudo-PD with MNI-PD in a single nonlinear warp estimate. After this process, refined pseudo-MRIs were defined based on the result of the first iteration and the procedure was repeated one more time. Registration between the histology-derived labels and the nonlinear template series was carefully inspected manually using 3D Slicer by three experts (KI, II, AH). Correspondence between histologic labels and MNI templates was evaluated and found to be very precise, especially but not exclusively in thalamic structures but in primary targets of functional neurosurgery (subthalamic nucleus, pallidum) as well.

Nomenclature and labeling used were in principle those of Walker (1938) with some modifications concerning motor nuclei proposed by Kultas-Ilinsky et al. (2011). In this system, VA defines the basal ganglia afferent territory that consists of two subdivisions, nigral afferent zone (VAn) and pallidal afferent zone (VAp). Cerebellar afferent territory is designated as VL. Within it, we marked its ventral region (VLv) that corresponds roughly to the part of the nucleus that was shown to display a high intensity staining for immunocytochemical marker SMI31 and is characterized by very large size neurons (Kultas-Ilinsky et al., 2011). It should be noted that individual subdivisions of some nuclei adjacent to motor thalamus have not been distinguished in the maps because in many instances the exact boundaries between them were not obvious in our material like in case of subdivisions of the mediodorsal nucleus (MD). Moreover, because of profuse interdigitation between centrolateral nucleus (CL) and the densicellular part of MD we indicated an approximate level of their transition from the rest of MD by a sharp change to a lighter density of the same color. Also due to very irregular boundary between centromedian (CM) and parafascicular (Pf) nuclei, the two were assigned the same color. In the region of somatosensory afferent projections that was designated collectively as VP the two subdivisions, medial (VPm) and lateral (VPl) were also assigned the same color as the transition from one to another was not quite clear, whereas the inferior subdivision (VPi) being quite distinct, was labeled differently (see color code in Fig. 2).

## Results

The focus of the study was on parcellation of functionally different territories of the motor thalamus and their topographic relationships, therefore only selected digital images of Nissl-stained sagittal sections and computer generated nuclear maps illustrating these relationships in three stereotactic planes are presented in this report. For the entire dataset containing also some nuclei and fiber bundles of the basal ganglia, subthalamic region, and midbrain, see www.humanmotorthalamus.com.

Successive mediolateral levels shown in Figures 3–7 illustrate the extent of the major territories of distribution of nigral, pallidal, and cerebellar afferent projections in the human thalamus.

Regarding the types of nerve cells and their distribution patterns in the homologous motor thalamic nuclei, there is no difference between human and rhesus monkey (Kultas-Ilinsky et al., 2011). In VL, large to medium size neurons are sparsely distributed and there are numerous groups of very small cells between them. The latter are GABAergic local circuit neurons described in earlier studies. In contrast, in VAp, varying size neurons, but none of them as large as in VL, are found in groups separated with passing through fiber bundles. Very small cells like the ones in VL are few. Thus, cytoarchitecturally, VL and the pallidal part of VA that runs along VL for a considerable distance are distinct. This can be seen in histology images in the left columns of Figures 3–7. The same is true for the boundary between the two VA subdivisions although this may not be very obvious in the low magnification histologic images demonstrated here. Unlike VAp cells, the neurons in VAn are large and darkly stained, but in contrast to VL neurons, they are found in groups. The common feature of VAp and VAn is the rarity of very small cells. In the dorsolateral part of VAn, the large neurons are smaller than in its ventromedial part but all other features are similar. Likewise, large neurons of the dorsal VL are smaller than those in its ventral part.

**Figure 8.**
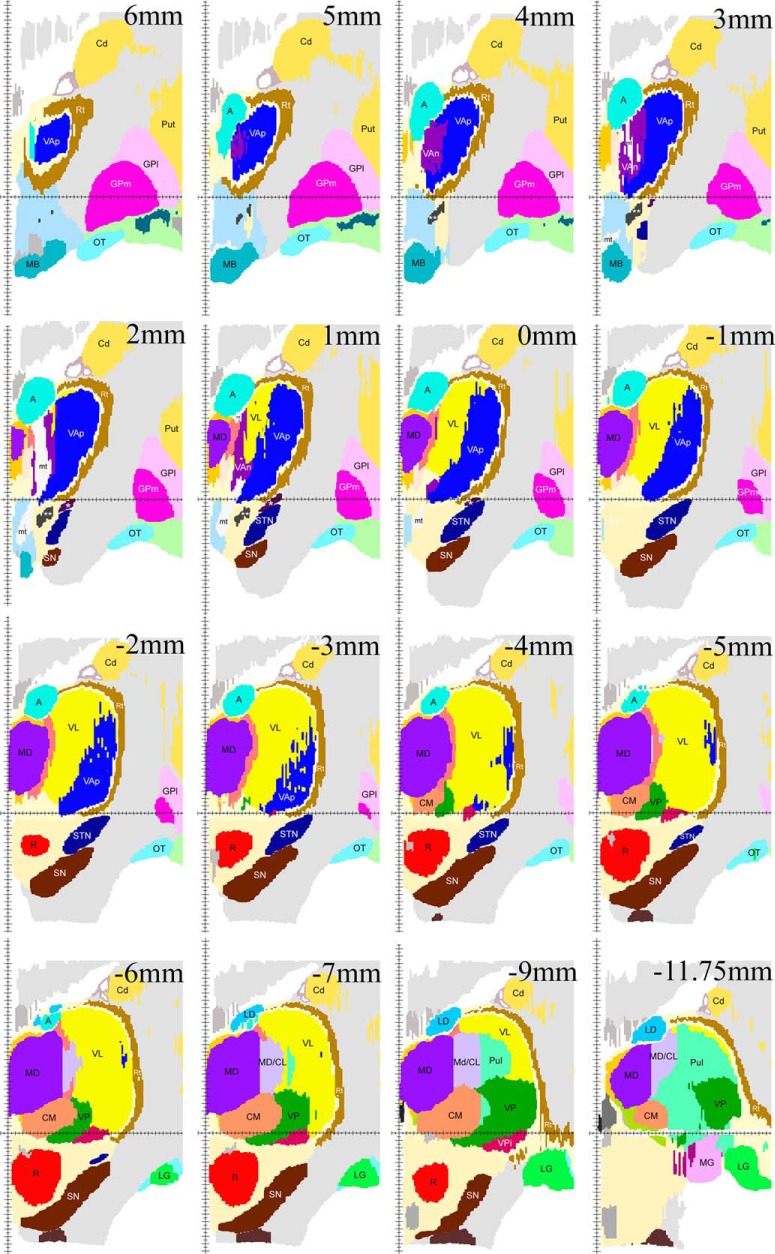
Examples of coronal maps derived from the reconstructed 3D volume. The cut at zero coronal plane (0 mm) is shown in the second image from the right in the second row. Cuts posterior to the zero coronal plane are marked with negative (-) numbers. Vertical axis indicates the midsagittal plane, horizontal axis indicates the zero horizontal plane. Because of low magnification of the images in this plate, pallidothalamic fiber bundles are marked as follows: lenticular fasciculus, one white asterisk; ansa lenticularis, two white asterisks; mt, mammillothalamic tract, other labeling as in Figures 3–7.

The common VAp/VAn, and VAp/VL boundaries are very uneven with deep protrusions of cell groups of one nucleus into another. The type of material used and restrictions of image processing techniques did not allow to demonstrate all these details in the maps. Therefore, only some of this unevenness is obvious in the images of the right column of Figures 3–7 as well as in coronal maps in Figure 8.

The volumes of the three nuclei are shown in Table 1. The largest territory in the motor thalamus is occupied by cerebellar fiber terminals, the nigral afferent territory is the smallest. The volumes of the three motor nuclei seem to be proportional to the sizes of their afferent sources. We also included for comparison the volumes of substantia nigra and medial globus pallidus in Table 1. It should be noted that nigral afferents to the thalamus originate only from pars reticularis of substantia nigra, thus the total volume from which the nigrothalamic fibers originate is roughly two thirds of that shown in Table 1. We do not have an appropriate figure for the volume of the deep cerebellar nuclei that are the source of cerebellar input to VL, but the massive size of the brachium conjuctivum (bc) seen in 4.75 and 6.00 mm histologic sections (Fig. 3) compared to the size of ansa lenticularis (al) and lenticular fasciculus (lf), also seen in several images of Figures 3–5, leaves no doubt about the larger mass of cerebellothalamic fibers compared to pallidothalamic.

Consistent with the volume size VL has the longest medio-lateral extent of 13.5 mm. Its most medial part is identifiable at 4.75 mm from the midline (Fig. 3) and it ends at ∼ 18.25 mm (Fig. 7). VAp is first present at 3.75 mm, but it extends only up to 16 mm from the midline. It should be noted that at the most lateral levels it is no longer in the form of a compact entity but as scattered cell groups embedded in VL. Only the largest of those is visible at 15.5 mm map in Figure 6. Groups of VAn neurons are identifiable slightly before 3 mm from the midline and no longer seen at 6.75 mm. The bulk of the nucleus is situated between 3.5 and 6.25 mm from the midline (Fig. 3).

The ventral region of VL (VLv) separated from the rest of the nucleus by a dashed line in the maps of Figures 4–6 was delineated based on high intensity of staining for cytoskeletal marker SMI31 in very large neurons (Kultas-Ilinsky et al., 2011). This region takes up approximately ventral one third of VL territory and extends roughly from 7 to 16.25 mm from the midline. Interestingly, the very large neurons of the ventral VL extend further to its medial most levels, but these do not display the very high intensity of SMI31.

**Figure 9.**
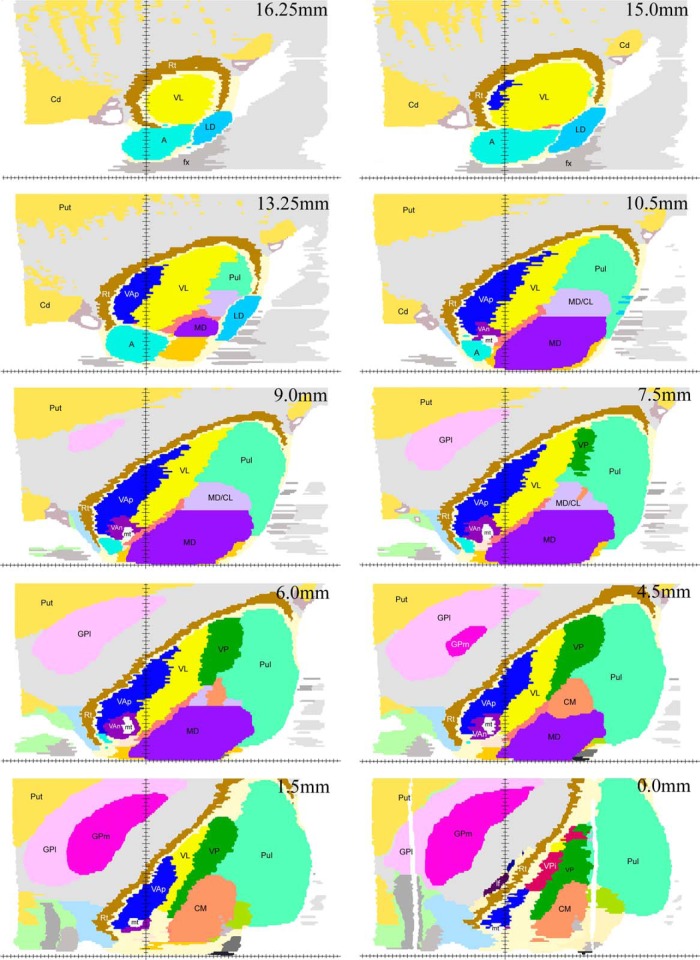
Examples of horizontal maps from the reconstructed 3D volume. All levels shown are above the zero horizontal plane as the motor nuclei do not extend below it. The cut at the zero level, i.e., at the AC-PC plane, is in the bottom of the right column (0.0 mm). Horizontal axis indicates the position of the midline. Vertical axis indicates position of the zero coronal plane.

The entire motor thalamic region is situated above the zero horizontal plane as seen in the right column of Figures 3–7, 8. Antero-posterior extent of the three nuclei varies. VAn is the shortest in anteroposterior and mediolateral dimensions and is entirely situated anterior to the zero coronal plane (Fig. 8). In contrast, the bulk of VL is situated posterior to the zero coronal plane. Its anterior-posterior dimensions vary depending on the dorsoventral coordinate. At medial levels, VL is narrow ventrally, ∼1 mm in width, whereas dorsally in the same sections, it is up to 4.0 mm in width (Fig. 3, bottom row). Laterally, the longest anteroposterior extent of the dorsal VL can be up to 13 mm (see 15-mm horizontal cut in Fig. 9). The longest anteroposterior extent of VLv is 4 mm at the lateral levels starting at ∼12.5 mm from the midline. VAp is situated both anteriorly and posteriorly to the zero coronal plane. At medial levels, dorsal part of VAp extends up to 7 mm anterior, while its ventral part extends up to 3.5 mm posterior, to the zero coronal plane at some lateral levels.

**Figure 10.**
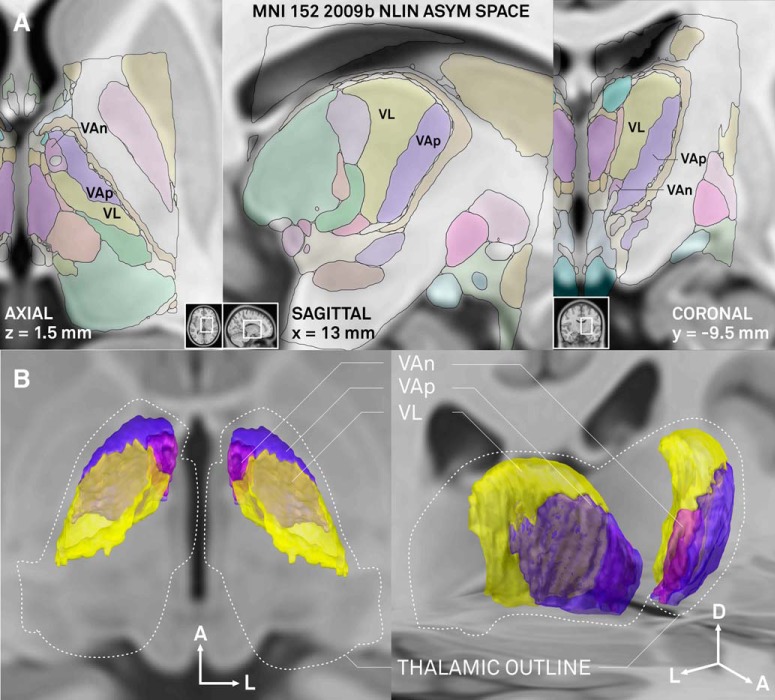
Illustration of overlap between coregistered atlas and MNI 2009b NLIN ASYM stereotactic space (T1 weighted template; Fonov et al., 2009). ***A***, Axial (left), sagittal (mid), and coronal (right) planes (positions of each plane in whole brain sections are outlined in corresponding insets). Basal ganglia (VAn, VAp) and cerebellar (VL) domains of the human motor thalamus are labeled, for other structures refer to color code in Figure 2 (please note that colors are less intense in this figure due to overlay on template). Coordinates marked on the panels are the ones used in MNI space, which are different from those used in the 3D atlas described here due to a difference in position of zero coronal plane. ***B***, Dorsal (left) and oblique (right) views of the basal ganglia and cerebellar domains as reconstructed in 3D. Outer thalamic outlines of the atlas are drawn with dashed white line. Arrows indicate orientation in the brain (A, anterior; L, lateral; D, dorsal).

The topographic relationships of the VAp and VL as well as their relationship to somatosensory complex is strikingly embodied in the horizontal plane. One can see in Figure 9 as well as in the axial (horizontal) plane of Figure 10*A* that pallidal and cerebellar territories, as well as adjacent somatosensory afferent area (shown in green) appear as consecutive bands offset relative to one another mediolaterally and at ∼45° angle to coronal plane. In contrast, the major axis of the nigrothalamic territory is vertical, as for the most part, it is aligned along the mammillothalamic tract (Figs. 3, 8).

Selected views from the MNI version of the atlas are shown in Figure 10. Correspondence of the structures to the T1 template may be evaluated in slices shown in Figure 10*A*. As can be seen, the fit to MNI space is in general very precise. It is very good for central structures (thalamus, subthalamic nucleus, medial and lateral globus pallidus as well as substantia nigra) but slightly worse for rim structures such as the striatum or mamillary bodies. The 3D view of subdivisions of the human motor thalamus is shown in Figure 10*B*. Shapes of single nuclei, spatial relationship between the three motor nuclei, and some other structures can be better appreciated in the Movie 1.

Movie 1. 3D relationships of motor thalamic nuclei and some other structures within MNI space. VL appears first followed by VAn and VAp. In the next sequence, all three appear together and then some other structures of particular relevance to deep brain stimulation such as medial and lateral globus pallidus, subthalamic nucleus, substantia nigra, and red nucleus are added. For unlabeled nuclei, refer to color-code in Figure 2. 10.1523/ENEURO.0060-18.2018.video.1

## Discussion

The main accomplishment of this study is a demonstration of the full extent of reliably identified human motor-related thalamic nuclei in three stereotactic planes derived from one brain. To our knowledge, this is the first demonstration of this kind. This report shows only selected images.

The entire section series through the thalamus and adjacent structures at 250-μm intervals with all nuclei present as well as supporting materials are available on a dedicated Internet site (www.humanmotorthalamus.com) linked to the Society for Neuroscience database (NIF; https://neuinfo.org). The MNI version of the atlas is available within Lead-DBS software www.lead-dbs.org.

In the present publication, we discuss only major subcortical afferent zones of the motor thalamus, namely nigral, pallidal, and cerebellar in the light of existing controversies and discrepancies as evidenced by publications in the fields of functional neurosurgery and brain imaging studies.

It is known from experimental studies in animals that fibers from medial globus pallidus, substantia nigra pars reticularis, and deep cerebellar nuclei reach also some other thalamic nuclei besides VAp, VAn, and VL. For example, there is some nigral and cerebellar input to MD (Ilinsky et al., 1985; Mason et al., 2000), but these projections are patchy and scanty, hence not easily identifiable in the human thalamus. Cerebellar output also reaches CL, but as pointed out above, its boundaries were indistinct in our material. On the other hand, in all species studied, pallidal input to CM is substantial and distributed to the entire nucleus. There is no compelling reason to believe that the same is not true for the human brain. Moreover, practically in all types of preparations the boundaries of CM are distinct, and at lateral levels very smooth, hence no controversies have been associated with identification of this nucleus.

The nuclear outlines illustrated here encompass only the major projection zones of nigral, pallidal, and cerebellar afferents to the human thalamus, as accurately as currently possible. However, some inaccuracies may be present at the most dorsolateral boundary of VL where it borders pulvinar because the transition from one nucleus to another was not very obvious in some sections due to compromised tissue quality.

Parcellation of primate thalamus based on a large variety of immunocytochemical staining patterns has been attempted in numerous studies. In our opinion, this has worked more or less successfully only for somatosensory afferent regions. Although the functional significance of predominance of one or another antigen in a specific region remained obscure, the staining patterns could be reliably correlated with modalities of peripheral somatosensory inputs (Rausell and Jones, 1991a,b; Rausell et al., 1992; Blomqvist et al., 2000). With respect to the motor thalamus, up until now, the correlations of distribution patterns of varying immunomarkers with subcortical afferent zones have not been conclusive, which has contributed its share to existing confusion in terminology and demarcations especially in the human (Morel et al. 1997; Jones, 2007; Morel, 2007). GAD65 staining patterns in the human motor thalamus described in detail by Kultas-Ilinsky et al. (2011) represented a step forward since they directly reflect the specifics of GABAergic circuits in each of the three subcortical afferent zones. Therefore, GAD65 staining may currently be considered as the most specific tool for demarcation of three functionally different areas of the motor thalamus in human. The nuclear maps presented here are based on these specific staining patterns, and together with proposed simplified function-related nomenclature, should provide a foundation for future parcellations within individual human motor nuclei using additional criteria when such become available.

We consider the nomenclature proposed here logical and straightforward hence easier applicable as compared to frequently referenced terminology proposed by Hirai and Jones (1989) and used in the atlases by Morel et al. (1997, 2007). In those publications, the pallidal afferent territory ended up being divided between two entities, VApc and VLa, despite there being no substantial difference between the two in either cytoarchitecture or various immunocytochemical staining patterns displayed. Hence, their outlines vary significantly in various anatomic publications, while distinction between the two subdivisions in imaging studies has become even more ambiguous. Moreover, in the above two publications, the dorsolateral part of the nigral afferent territory has completely vanished from the thalamus being apparently lost in pallidal afferent zones. These deficiencies were partially overcome by Mai and Forutan (2012), who, based on the results of their thorough immunocytochemical analysis of human thalamus, divided the anteromedial part of the motor thalamus in two subdivisions, VA lateral and VA medial, which roughly but not entirely correspond to our VAp and VAn, i.e., to pallidal and nigral territories respectively, whereas the territory they designated as VL in general coincides with the VL as defined in this study, that is cerebellar afferent territory.

The above mentioned revisions of nomenclature were set off by complexity of Hassler’s terminology that is used in the widely popular stereotactic atlas by Schaltenbrandt and Wahren (1977). In contrast, GAD65 staining patterns in the human thalamus allowed to relate individual Hassler’s nuclei to subcortical projection zones as summarized in Kultas-Ilinsky et al. (2011), their Table 3. In the present study (Table 2), we went one step further and removed semantic ambiguity from the nomenclature so that each subcortical afferent territory has a unique name. As shown in Table 2, each of the three motor nuclei encompass several Hassler’s nuclear entities. In consequence, the enigma of unknown functional roles of the dorsal thalamic subdivisions in Hassler’s maps (Doi, Zo, Doe, Dim, Zim, Zc) is eliminated. These Hassler’s subdivisions represent just a dorsal aspect of the basal ganglia or cerebellar projection zones. Thus, in the human thalamus, just as shown earlier in monkeys, motor-related nuclei extend all the way up to the dorsal boundary of the thalamus meaning that they are not confined only to its ventral part as it has been thought for some time. One may now assert that the dorsal thalamic nuclei of Hassler included in the three motor nuclei outlined here (Table 2) are involved in processing of motor-related information derived from the basal ganglia and cerebellum just as the subdivisions ventral to them. This, of course, does not exclude the possibility of fine functional differences between dorsal and ventral parts in some or in all three motor nuclei, which may be due to fine differences in their cortical connections or nuances of modalities processed.

**Table 2.** Comparison of classifications of human motor-related thalamic nuclei of this study and that of Hassler (1959)

Subcortical afferent sources	Nuclear subdivisions of this study	Corresponding nuclear subdivisions of Hassler
		
Substantia nigra pars reticularis	VAn	Lpo.mc and parts of Lpo and Doi
Medial globus pallidus	VAp	Voa, marginally Vop, parts of Lpo, Doi, Zo, Doe, and Voi
Deep cerebellar nuclei	VL (VLd and VLv)	Vim, bulk of Vop, and parts of Voi, Vom, Dim, Doi, Zim, Zo, and Zc

Extensive discussion in earlier neurosurgical literature was devoted to identification of the most effective thalamic target of stereotactic interventions for elimination of tremor (Ohye and Narabayashi, 1979; Laitinen 1985; Hirai et al., 1989; Lenz et al., 1990). The debate focused on whether the effective locus was in Hassler’s Vim or Vop, at that time presumed to be cerebellar and pallidal projection zones, respectively, or in both (for review, see Hamani et al., 2006). On the other hand, the GAD65 staining pattern in the area outlined as Vop in Hassler’s maps is identical to that in the area outlined as Vim, indicating that both are part of the cerebellar afferent territory except for a narrow strip at the anterior end of Vop, which together with Voa displays staining pattern characteristic for pallidal afferent territory (Kultas-Ilinsky et al., 2011). Therefore, both Vim and the bulk of Vop of Hassler are a part of VL under nomenclature applied here (Table 2), thus confirming earlier suggestions that both subdivisions represent cerebellar afferent zone (Ilinsky and Kultas-Ilinsky 2002a: Krack et al., 2002). Moreover, Vim and Vop together form the VLv as marked in the maps illustrated here. According to Lenz et al. (1995), the most effective target for eliminating tremor is located in the area between 14 and 15 mm laterally, 2 mm anterior to the VP, and 3 mm above AC-PC line. As seen in the sagittal maps of Figure 6, this location is well in VLv and does not impinge on VAp. Coordinates of Vim listed in the probabilistic functional atlas by Nowinski et al. (2005) measured from the anterior end of posterior commissure also fit nicely in our VLv, although they differ from those of Lenz in dorsoventral coordinate. Likewise, the positions of tremor cells illustrated in the diagrams of Brodkey et al. (2004) also fall well within VLv as outlined here. Moreover, in view of very uneven boundary between the VL and VAp some of the points in the diagrams that scatter anterior to Vim may not necessarily be in the pallidal territory. Thus, it seems to us that finally the issue of Vim versus Vop can now be considered solved.

As described in Results, the topographic relationships of pallidal, cerebellar and adjacent somatosensory territories in the thalamus when viewed in horizontal cuts represent three consecutive anteroposterior bands, with their width depending on the dorsoventral coordinate, and tilted at ∼45° relative to coronal and midsagittal planes. Comparing this topography with some published probabilistic maps of the thalamus constructed with diffusion tensor imaging (DTI) techniques that rely on cortical connectivity (Johansen-Berg et al., 2005; Klein et al., 2010; Kincses et al., 2012; Mang et al., 2012), one can see that while the anteroposterior sequence of the zones is similar, their neuroanatomical orientation, shapes, and relative sizes differ in the two datasets. The most striking and substantial difference is that the zones in those probabilistic maps are oriented mediolaterally at an almost 90° angle to the midsagittal plane implying that they only partially overlap with subcortical afferent territories and do not accurately follow the distribution of terminals of corticothalamic fibers as known from experimental studies in nonhuman primates. The simplest explanation is the difference in the spatial resolution of the neuroanatomical and DTI techniques. DTI reveals mainly the fiber bundles whereas anatomic pathway tracing and specific immunostaining patterns expose the terminal zones. Besides, both cortical and subcortical fiber bundles on entering thalamus break up to individual fibers that take sometimes very tortuous course before reaching their final destinations (see for example individual cerebellothalamic fibers in Mason et al., 2000; and corticothalamic fibers in Kultas-Ilinsky et al., 2003). The exception are the prefrontal cortex fibers running in the anterior limb of internal capsule that stay within the bundles for some distance in the thalamus specifically when passing through VA, i.e., the basal ganglia afferent zone, en route to MD. These fibers appear to be responsible for the large size of prefrontal cortex territory in the thalamus as demonstrated in most of published DT images that show it occupying the frontal one third of the thalamus throughout its entire mediolateral extent but not extending to its posterior aspect, i.e., to MD nucleus where the prefrontal cortex efferents terminate. Thus, the maps of the thalamic nuclei in three stereotactic planes demonstrated here as well as the full sets of atlas plates available separately provide the common ground on which the correlation between the results of probabilistic and deterministic DT imaging studies on one hand and neuroanatomical experimental data on another can be built.

Above and beyond demonstrating clear afferent domains in the human motor thalamus in volumetric fashion, the atlas established in the present study can be of great use for neuroimaging studies as well as stereotactic surgery, based on the possibility to nonlinearly register our atlas to individual brains via the use of the MNI template. In case of the former, results of fMRI studies on task activation or diffusion-weigher tractography studies may be assigned to finer subregions (Behrens et al., 2003). In case of the latter, the stereotactic method is slowly migrating from ACPC-based coordinate system toward the use of nonlinear MNI coordinates (Horn et al., 2017a). The ability to warp brain atlases to individual patient brains in a precise fashion yields strong potential for surgical planning (Mayberg et al., 2005; Riva-Posse et al., 2017). Finally, registration to brain atlases is already being applied to guide programing of deep brain stimulation impulse generators after surgery (Horn and Kühn, 2015; Husch et al., 2017; Horn et al., 2017b). For such applications, the present atlas depicts the most efficient and practical solution. In comparison to Hassler’s outlines and terminology or the Hirai and Jones’ nomenclature, the present parcellation focuses on the clinically most important and accurate information needed on subparts of the movement-related projection system to the human thalamus.

## Synthesis

Reviewing Editor: Menno Witter, Norwegian University of Science and Technology Decisions are customarily a result of the Reviewing Editor and the peer reviewers coming together and discussing their recommendations until a consensus is reached. When revisions are invited, a fact-based synthesis statement explaining their decision and outlining what is needed to prepare a revision will be listed below. The following reviewer(s) agreed to reveal their identity: Jose Lanciego, Edward Bertram.

This is a nice anatomical study providing an updated parcellation together with a simplified nomenclature of the thalamic nuclei and adjacent structures. The authors provide a more precise subdivision of components of the human thalamus, with emphasis on the motor thalamus based on differences in GAD65 immunostaining of human brain sections. The immunostaining was mapped onto the nuclei that were determined by Nissl staining. Based on the used sagittal sections, the authors managed to provide coronal and horizontal -axial- projections ultimately leading to a 3D atlas that incorporates stereotaxic coordinates based on the ac-pc plane. Being constructed this way; this 3D atlas is co-registered with the MNI-152 template space. Unfortunately. the paper fails partially in achieving the stated goal to help stereotactic localization for neurosurgical procedures in which more precise spatial placement is needed. For this, it would be important to deal with that fact that the studies were performed on the brains from people without defined neurological disease. As most neurosurgical procedures targeting the thalamus are because of a disease (e.g., various movement disorders often associated with degeneration), it is not clear how the relationships might change in various disease states and how placement may require changes in placement coordinates. Adding internal landmarks visible on imaging studies that would serve as a guide for final stereotactic coordinates is essential. Without clear internal landmarks, visible on a clinical MRI scan, limits the application of these data for the intended clinical purpose. i.e., there is no certainty that the placement of surgical interventions will be functionally close to the intended target.

The paper however does achieve the other goal to assist in the interpretation and presentation of data derived from functional MRI and DTI studies, which is in line with many such 3D generalized space projects in animal neuroscience as well. This usage is facilitated since the generated data have been made publicly available under an open license.

One can easily appreciate the enormous effort taken during the preparation of this contribution. This kind of work requires strong histological skills and deep knowledge of tissue processing, a very demanding issue particularly when dealing with the human brain. This is best exemplified throughout lines 119-127 (section Materials and Methods), where the authors explained why they have chosen sagittal sections as the backbone of the conducted work.

Overall, the manuscript is well-written, pleasant to read and nicely illustrated. We have following suggestions for a revision:

1. Either add internal MRI landmarks or downplay the potential clinical use of the paper (see also the next comment).

2. Although providing a 3D space atlas comprising a full segmentation of thalamic nuclei is the main aim of this contribution, a number of key adjacent structures are also delineated within the illustrated segmentation throughout all enclosed figures. This is fine to me, however, I guess that a more detailed parcellation of several structures should also be included in a renewed version. For instance, bearing in mind that this contribution would be of instrumental value for stereotaxic procedures targeting deep brain structures related to movement disorders, I would strongly encourage the authors to pay more attention to the segmentation provided for the subthalamic area. This area is a bottleneck of deep interest for functional neurosurgery approaches, and comprises a hub through which several pathways and fasciculi are coursing through. Even if the parcellation is based on Nissl stains, structures like the Forel's fields H1 and H2 (ansa lenticularis, fasciculus lenticularis and thalamic fasciculus) should be included within the provided segmentation. This also applies to structures lying in between the subthalamic nucleus and the thalamus such as for instance the zona incerta. Furthermore, please note that the mamillothalamic tract (mtt) is a key reference for STN-DBS since this tract represents the mid-point for the ac-pc line. Here, the mtt is only illustrated in the top illustration of Figure 3 (taken at 3.25 mm away from the midline) and it is very hardly visible in all the provided coronal and horizontal reconstructions. In summary, by providing a better segmentation of structures within the subthalamic area then this contribution would undoubtedly gain an important added value.

3. Figures 8 and 9 comprise representative maps of coronal and axial projections gathered from sagittal sections. Segmented structures and nuclei were color-coded. Although color codes were probably chosen here in an attempt to minimize maps overcrowded with press-on-lettering, going back a forth constantly to the color codes list is somehow a monotonous procedure. I guess that adding some abbreviations here illustrating the obtained projected parcellation could make sense.

4. Figure 10 provides some examples of co-registration between the MNI template space and the related parcellation across all axis x, y and z. Although this is fine to me, perhaps adding a few more examples taken at lower magnification might be helpful for non-experienced users.

5. When going through the significance statement (lines 27-28), the authors quoted that the atlas was generated “from a continuous series of histological sections from a single brain”. However, later on within the Materials and Methods section, the use of four postmortem human brain specimens (two males and two females) is described. I guess that this apparent discrepancy should be better explained.

6. Finally, you should include additional relevant references on stereotaxic atlases. When dealing with macaque studies, please include the stereotaxic atlas made by Lanciego and Vazquez (Brain Struct Funct 2012). Human brain atlases published by Ann Morel (2007) and by Jürgen Mai and co-workers (2008) should also be listed.
